# Taking Others' Perspectives Enhances Situation Awareness in the Smart Home Interface

**DOI:** 10.3389/fpsyg.2019.02761

**Published:** 2019-12-10

**Authors:** Sanghyeong Yu, Kwanghee Han

**Affiliations:** ^1^Laboratory of Cognitive Engineering, Graduate Program in Cognitive Science, Yonsei University, Seoul, South Korea; ^2^Laboratory of Cognitive Engineering, Department of Psychology, Yonsei University, Seoul, South Korea

**Keywords:** visuospatial perspective taking, situation awareness, internet of things, smart home, affordance

## Abstract

In the smart home environment, all devices are connected to each other and are shared by co-users living together. This may make people's interactions with the devices more complicated, owing not only to the difficulty of meeting each co-user's tastes with respect to how the devices operate, but also to variations in the frequency of device use among family members. If so, it is inevitable that using multiple devices by multiple users can lead to difficulty maintaining situation awareness. Therefore, to relieve such interaction problems caused by the presence of co-users, we examined the effect of spontaneous visuospatial perspective taking on situation awareness with respect to the smart home interface. To this end, we measured whether merely the affordance of other users can elicit spontaneous visuospatial perspective-taking, replicating previous research. We also examined whether the affordances of other users can help enhance situation awareness in the mock-up smart home interface design we created. When participants adopted affordances of other users' perspectives, they could easily perceive the information about the devices. However, when they viewed the devices from other's perspective, their understanding of devices mainly used by the self-remained relatively low. Potential reasons for these findings are discussed along with proposals for future research.

## Introduction

The emerging Internet of Things (IoT) requires us to think about human-computer interaction (HCI) from a new perspective. Traditional HCI research has long investigated how we design interactions using a single device (e.g., personal computer, mobile phone) by a single person. However, the IoT —a new type of interaction—is the interaction involving multiple users with multiple devices that are connected to each other (Ashton, 2009). Thus, we need to consider the relationships among many users and the context between the connected devices for design purposes (Cila et al., 2017; Cervantes-Solis, 2019). For example, in the smart home—which means that the IoT is installed in the house—we can control multiple devices simultaneously through a controller. Usually, the device is a mobile application, which helps users to understand the situation and to do their household or routine work (Jakobi et al., 2017). Specifically, Amazon Alexa—an AI speaker mobile application which is also a smart home controller—has the *routine* menu including features such as *sleep mode*, a situational mode that allows turning off the TV or dimming the lights at a specific time. In addition, Google Assistant—another AI speaker mobile application that is also a smart home controller—makes users identify the device's status and control the devices based on their room. As such, the emergence of the IoT may cause an interaction paradigm shift that would make traditional HCI disappear (Console et al., 2013; Rapp et al., 2019). Therefore, we need to cope with interaction problems that may arise owing to the difference between traditional HCI and IoT.

Above all, sharing across devices by co-users living together may cause a complicated interaction within the IoT. Even if they share the same devices, different users may have different needs with respect to when and how the devices work. For example, some users may want the light of a lamp currently beside them to be lime-colored. However, that lamp may have previously been programmed to turn blue at bedtime by other users. Likewise, the configuration of the devices and how the devices operate considering the daily routine—situational modes such as *Home mode, Away mode, Wake-up mode*, and *Sleep mode*—can differ across family members. Unfortunately, the smart home controller interface does not currently support variations across co-user's tastes or preferences.

Furthermore, the fact that the frequency of device use varies across family members may increase the complexity of the interaction. Each device's primary user is not always the same person—the primary user can be various family members. For example, even if I rarely use the vacuum cleaner, other family members might use the vacuum cleaner almost every day. However, sometimes we use the vacuum cleaner because it is common for family members to ask one another to do others' housework when they are busy or absent. As such, when using devices I seldom use, it can be difficult to understand what the status of the device is and which functions are reserved. In other words, it may be hard to maintain situational awareness (Endsley, 1995) about the smart home controller interface when using infrequently used devices. According to Endsley (1995), situational awareness is the understanding of dynamic system interfaces. Specifically, situation awareness follows three steps: Level 1 is the *perception* of the interface elements, Level 2 is the *understanding* of the interface elements, and Level 3 is the *projection* based on the understanding of the interface. Returning to the fictitious case of the vacuum cleaner I rarely use, it can be hard to understand where the vacuum cleaner is located (i.e., Level 1), what the status of the vacuum cleaner is (i.e., Level 2), and how the vacuum cleaner will be operated in the future (i.e., Level 3).

Such interaction problems caused by co-users could be solved by understanding affordances in the smart home interface. Affordances refer not only to the interpretation of an object or environment (Norman, 1999) but also to inducing a user's behavior in pursuit of a shared intention and goal through understanding the interaction (Baber, 2018). An advantage of this affordance is that it can make users automatically and tacitly perform certain behaviors with little mental effort (Grgic et al., 2016). If we design optimized affordances for the smart home environment, it would help users to better encode and retrieve information about smart home interactions. For example, even if the device is not frequently used by some users, those users could take advantage of high situation awareness through intuitive affordances of co-users—such as icons—in the smart home interface. The affordances of co-users can be effective for helping users think about interactions from the perspective of those users.

We sometimes look at or think of an object from others' perspectives owing only to the mere presence of those others. According to Tversky and Hard (2009), it is natural to take an egocentric reference frame, which refers to viewing the world through the perspective of the self. However, numerous recent studies have revealed that we adopt the visuospatial perspective of others when sharing physical space with others (Tversky and Hard, 2009; Kockler et al., 2010; Freundlieb et al., 2016; Furlanetto et al., 2016; Cavallo et al., 2017; Quesque et al., 2018). For example, we may explain the location of an object based on the perspective of another person sitting across from you by referring it to it as “the apple on your left” instead of “the apple on my right” (Cavallo et al., 2017). This is called the spontaneous visuospatial perspective taking (i.e., VSP taking; Tversky and Hard, 2009; Freundlieb et al., 2016, 2018; Cavallo et al., 2017). This VSP taking is divided into two levels. Contrary to VSP taking level 1 that refers to whether other people can see an object or not (Flavell et al., 1981; Samson et al., 2010; Furlanetto et al., 2016) VSP taking level 2 focuses on how an object is shown from others' point of view, as mentioned above. Recent findings show that such VSP taking level 2 may not only be limited to the physical realm but may also extend to mental activity such as word reading (Freundlieb et al., 2018). According to Freundlieb et al. (2018), this propensity to adopt other peoples' VSPs can help to create shared meaning and facilitate information processing. Thus, if we adopt another person's perspective, we may perceive and understand the smart home interface—especially devices we rarely use—as if walking in others' shoes.

Therefore, we examined whether the affordance of other users' perspectives can enhance situational awareness about the smart home interface in two experiments. In Experiment 1, we aimed to measure whether spontaneous VSP taking can occur solely through the affordance of other users. In Experiment 2, we examined whether the affordance of other users' perspectives not only causes spontaneous VSP taking but enhances situation awareness in a mock-up design of a smart home interface.

**Figure 1:**
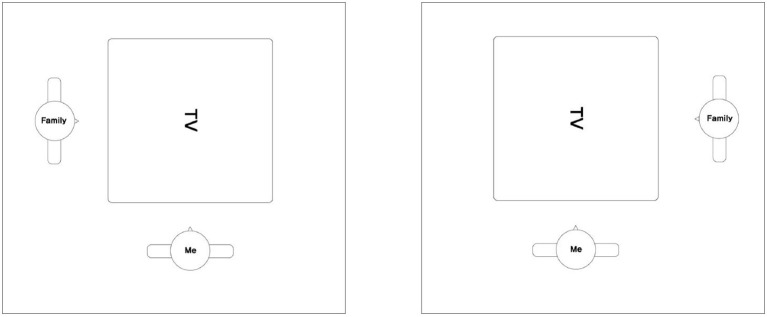
Stimulus in Experiment 1. A stimulus word “TV” rotated 0 degrees from the other's perspective in congruent condition **(Left)** and rotated 180 degrees from the affordance of the other' perspective in incongruent condition **(Right)**.

To this end, we adopted a previously used paradigm showing VSP taking in mental space (Freundlieb et al., 2018). In that experiment, participants categorized a word from two categories (i.e., animals and vegetables/fruits) that was always displayed vertically—rotated 90 degrees—from the perspective of a participant (i.e., self-perspective), as shown in Figure 1. From the perspective of others, the word was placed at 0 degrees (i.e., congruent condition) or rotated 180 degrees (i.e., incongruent condition). According to Aretz and Wickens (1992), angular disparity can make it take longer to read a word. Therefore, it may take the least amount of time to read words at 0 degrees (i.e., congruent condition), and then 90 degrees, and finally 180 degrees (i.e., incongruent condition) owing to the discrepancy of the mental rotation. As such, if participants take their own perspective, there will be no difference in time to read between the congruent and incongruent condition; however, if they adopt others' perspectives, it takes less time to read a word in the congruent condition and more time in the incongruent condition. If a participant takes the perspective of others, they should categorize the words faster and more accurately in the congruent condition as compared to their own perspective. As mentioned previously, that result can be explained by *congruency effects* where the congruent condition—a match between the other's perspective and the word direction—leads to better performance than the incongruent condition.

In Experiment 1, the previous experiment (Freundlieb et al., 2018) was modified to make it more suitable for a smart home environment. While the original experiment used stimulus words from two categories (animals and vegetables/fruits), we used a word list referring to smart home devices. The words were divided into two categories (self and other user) depending on the primary user of the device. In addition, unlike the previous experiment with an actual person (i.e., confederate) in physical space, the current study created a virtual shared physical context (stimulus image and mock-up interface) by displaying the affordance of other users, affordance of the self, and words (i.e., smart devices) on the computer screen (as shown in Figure 1). If this experiment replicated the results of the previous experiment, participants may categorize more quickly and accurately in the congruent condition where the affordance of another user's perspective and the words match.

In Experiment 2, we designed the mock-up application for the smart home to make the experiment environment more similar to actual smart home context. On the top of the mock-up design of the smart home interface were same stimulus images as in Experiment 1, including the affordance of the other user, affordance of the self, and the device words. On the bottom of that smart home interface was the status of devices and further information of situational modes. We assumed that if VSP taking occurred in the smart home interface, the affordance of the other user would help participants maintain high situational awareness.

## Experiment 1

### Materials and Methods

#### Participants

Sixty-four undergraduates from Yonsei University participated in this experiment (aged 21–26 years, *M*_age_ = 22.24 years, *SD*_age_ = 1.78 years; 32 women). All participants received course credit as compensation. Participants provided written consent, and all procedures were conducted in accordance with Code of Ethics of the World Medical Association (Declaration of Helsinki). Experimental procedures were approved by the Institutional Review Board of Yonsei University.

An *a priori* power analysis (Faul et al., 2007) using an effect size of *f* = 0.2, α = 0.05, and 1 – β = 0.95 indicated that data from 55 subjects should be collected. We recruited nine additional participants to prepare for outliers exceeding 2 *SD* from the mean reaction time.

#### Design

A repeated-measures analysis of variance (ANOVA) was selected as the analysis technique. The independent variables were the congruency between the affordance of the other user's perspective and the word (2: congruent, incongruent) and the primary user-specific devices (2: self, other). The dependent variable was the time it took to categorize the smart home device words accurately depending on the primary user of the device.

#### Task

The current study was based on a previous experiment that revealed that VSP taking occurs in mental space (Freundlieb et al., 2018). In Phase 1, participants were instructed to memorize the 16 smart home device words (see Table 1), which were divided into two categories depending on the primary user (e.g., self, other) who frequently uses that device. Among them, eight words referring to the smart home devices were frequently used by the self and eight were mainly used by the other. Participants were asked to memorize the 16 devices to focus on who is the primary user.

**Table 1:** List of primary user-specific smart home devices in Experiment 1.

**Self**	**Other**
Air conditioner	TV
Refrigerator	Air cleaner
Lamp	AI speaker
Robot vacuum cleaner	Multi-tap
Washing machine	Dish washer
Dehumidifier	Smart oven
Home CCTV	Blind
Door Sensor	Smart plug

In Phase 2, there were evaluated on whether they memorized the smart home devices and the primary users. After a word referring to the smart home device (e.g., TV, lamp, and robot vacuum cleaner) appeared for 1,500 ms on the computer screen, the following screen displayed a word indicating the primary user (e.g., self or other). If the user displayed on the screen is the primary user of that device, participants were instructed to press the *Yes* button. If not, they should press the *No* button. This screen disappeared when users answered.

Given that categorizing those smart home devices into self or other categorizes can be more difficult than those in previous experiment. Because the stimulus words of current study are about smart home devices, which could be put in the same category. Therefore, we offer participants time to memorize in phase 1 and phase 2. Participants memorized the written list of smart home devices word on paper in phase 1 and they could check whether their response to who the device's primary user is correct through the computer screen in phase 2. The data of participants with a memory accuracy of <90% will be excluded from the analysis.

Last, in Phase 3, participants should respond with respect to who is the primary user of the smart home device just after reading a word related to the smart home device on the computer screen. The stimulus image included the affordance of the self, affordance of the other, and a device word. A stimulus word was always displayed rotated 90 degrees from the affordance of the self's perspective in all conditions; however, from the affordance of the other's perspective, device words rotated 0 degrees were in the congruent condition, and words rotated 180 degrees were in the incongruent condition.

#### Procedure

Participants were requested to memorize the list of primary user-specific smart home devices for 10 min in Phase 1 (see Table 2). In Phase 2, participants viewed smart home device words on the computer screen for 1,500 ms. After this screen, participants read a primary user word (self or other) located in the center of the slide until a response was made.

**Table 2:** Procedures of Experiment 1.

**Phase 1**	**Phase 2**	**Phase 3**
Memorize the list (10 min)	Memory test for the list of Phase 1 (5 min)	Device categorization task with affordances (10 min)

All participants were asked to respond to whether the *user* (i.e., self or other) appearing on the screen was the primary user of the device written on the previous screen. If the *user* is the primary user of the device, participants should press “1” with sticker “Y” on it. If the user is not the primary user, they should press “0” with sticker “N” on it. A smart home device word appeared in 48 trials, consisting of three repetitions of 16 trials.

In Phase 3, the stimulus image (see Figure 1) was a smart home device word in the middle of two affordances, and participants were requested to specify the primary user of that smart home device. The participants were told that the human agent is a human figure that face to the device word. If participants thought that they were the primary user of that smart home device, they pressed “g” with sticker “ME.” If they thought the primary user was someone else, they pressed “j” with sticker “FAMILY.” To match the meaning of the image and the stickers, we used the “ME” and “FAMILY” stickers instead self and other. There were 128 trials in total: 64 in the congruent condition and 64 in the incongruent condition.

### Result and Discussion

All participants showed >90% accuracy in the memory task in Phase 2. Therefore, all participants' data were used in the analysis except for the data (2.89%) of error trials and reaction times (RTs) more than 2 *SD*s (4.69%) from each participant's condition mean.

We conducted a 2 (congruency with other's perspective: congruent, incongruent) × 2 (primary user-specific device: self, other) repeated-measures ANOVA for the results in Phase 3 (see Table 3). There was no significant difference [*F*_(1, 63)_ = 0.011, *p* = 0.918, η^2^p = 0.000] between the congruent (*M* = 901.234, *SD* = 146.936) and incongruent condition (*M* = 902.675, *SD* = 137.767). However, there was a main effect for the primary user-specific device condition [*F*_(1, 63)_ = 10.458, *p* = 0.002, η^2^p = 0.142]. When the primary user of that stimulus device word was the other user (*M* = 921.449, *SD* = 141.091), it took longer to judge who is the primary user of that device than it did in the self-user condition (*M* = 883.788, *SD* = 155.160).

**Table 3:** A 2 (congruency with others' perspective: congruent, incongruent) × 2 (primary user-specific device: self, other) repeated-measures ANOVA in Experiment 1.

**Source**	**df**	***F***	**η^2^**	***p***
Congruency	1	0.011	0.000	0.918
Primary user-specific device	1	10.458	0.142	0.002
Error	63	(191.001)		
Congruency × primary user-specific device	1	1.045	0.016	0.311
Error	63	(3306.320)		

*Values enclosed in parentheses represent mean square errors*.

**Figure 2:**
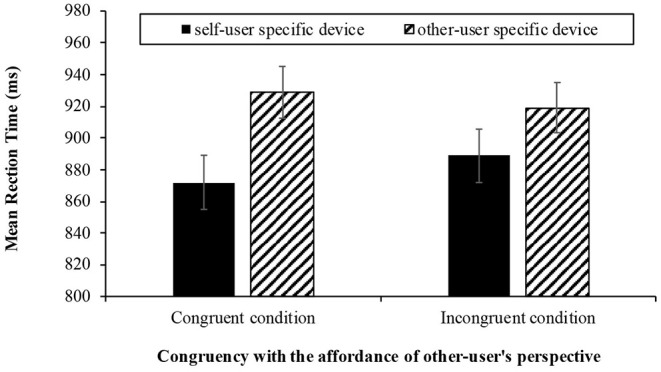
The results of mean reaction time (± *SE*) it took to categorize the smart home device words accurately as the congruency between affordance of the other user's perspective and the word (2: congruent, incongruent) and the primary user-specific devices (2: self, other) in Experiment 1.

In addition, there was no interaction between congruency and primary user-specific device [*F*_(1, 63)_ = 1.045, *p* = 0.311, η^2^p = 0.016; see Figure 2]. Specifically, the mean reaction time was lowest for the self-user specific device condition and congruent condition (*M* = 879.684, *SD* = 163.971), the self-user specific device condition and incongruent condition (*M* = 887.598, *SD* = 154.805), the other-user specific device condition and incongruent condition (*M* = 917.527, *SD* = 142.989), and finally, the other-user specific device condition and congruent condition (*M* = 924.306, *SD* = 145.455).

In conclusion, the result of Experiment 1 did not replicate the previous experiment (Freundlieb et al., 2018). We only found a main effect of the primary user-specific device (i.e., category effect). In that previous experiment, there was no category effect—that is, there was no difference in mean reaction time between the two categories. However, the results of Experiment 1 revealed a category effect according to who is the primary user of the smart home devices (self or other).

The category effect may have emerged here because the stimulus words (i.e., the smart home devices) in the current experiment were different from the stimulus words (i.e., the animals and vegetables/fruits) used previously. The stimulus words referring to smart home devices were more related to the self and other people as compared to animals and vegetables/fruits. Animals and vegetables/fruits are independent of people (i.e., self and others); however, in Experiment 1, both stimulus words and affordances divided into self and other were related to people. Thus, rather than eliciting a spontaneous VSP taking phenomenon, participants might have engaged in more elaborative cognitive processing to recognize and classify the self and others upon reading the stimulus words.

In addition, the way of presenting the human agent may also have affected the category effect. While there was an actual human (i.e., confederate) in the previous experiment, there was a human figure (i.e., affordance of self and other) on display in the current experiment. We initially expected that this difference would not affect the VSP taking phenomenon because this phenomenon also occurred in non-human agent features such as an arrow and triangle. However, given that the congruency effect—reading stimulus words using the affordance of other's perspectives— did not occur in Experiment 1, the human figure that looks like a human but is not a real person might be not suitable to induce VSP taking than an arrow or triangle.

Otherwise, these results might be due to participants' memory strategy in which they memorize self-user specific device more intensively. Participants categorized self-user specific device faster than other-user specific devices regardless of whether they were in the congruent or incongruent condition. If participants use a strategy of memorizing one of the two categories (e.g., self and other), the majority may have chosen their own category than others.

However, above all, we thought the congruency effect may appear if the experimental environment better approximates the actual smart home interface. This is because the simplicity of the experimental environment in Experiment 1 could make participants focus more on stimulus words. The stimulus words may have been too salient to adopt others' perspective and this salient stimulus words may contribute to executing participants' memory strategy rather than VSP taking. This saliency of stimulus words may be relieved by other important information which is needed to be processed in an actual smart home interface. Therefore, we thought that participants may not be affected by stimulus words such as the primary user-specific device, if we made a complicated experimental environment similar to an actual smart home interface.

## Experiment 2

We examined whether participants adopted the affordance of others' perspectives in an experimental environment similar to a real smart home. To this end, we designed a mock smart home mobile application interface. In that mock-up design interface, there is information about the smart home devices such as location, status, and situational reservation functions (i.e., situational mode).

All the participants completed the Situation Awareness Global Assessment Technique (SAGAT) (Endsley, 1995, 2017; Scholtz et al., 2005) after watching the mock-up of the smart home mobile application interface. We assumed that if participants adopted the affordance of the other user's perspective, their situation awareness may be enhanced in the congruent condition (i.e., a matching between the affordance of the other user's perspective and direction of stimulus words).

### Materials and Methods

#### Participants

Sixty-four undergraduates from Yonsei University participated in this experiment (aged 19–26 years, *M*_age_ = 21.75 years, *SD*_age_ = 2.94 years; 40 women). As in Experiment 1, all participants received course credit as compensation. They provided informed written consent, and all procedures were approved by the Institutional Review Board of Yonsei University and conducted in accordance with the Code of Ethics of the World Medical Association (Declaration of Helsinki).

*A priori* power analysis was the same as in Experiment 1.

#### Design

The independent variables were same as in Experiment 1. The dependent variables were response accuracy for the situation awareness (SA) Level 1, 2, and 3 evaluation questionnaires.

#### Task

**Figure 3:**
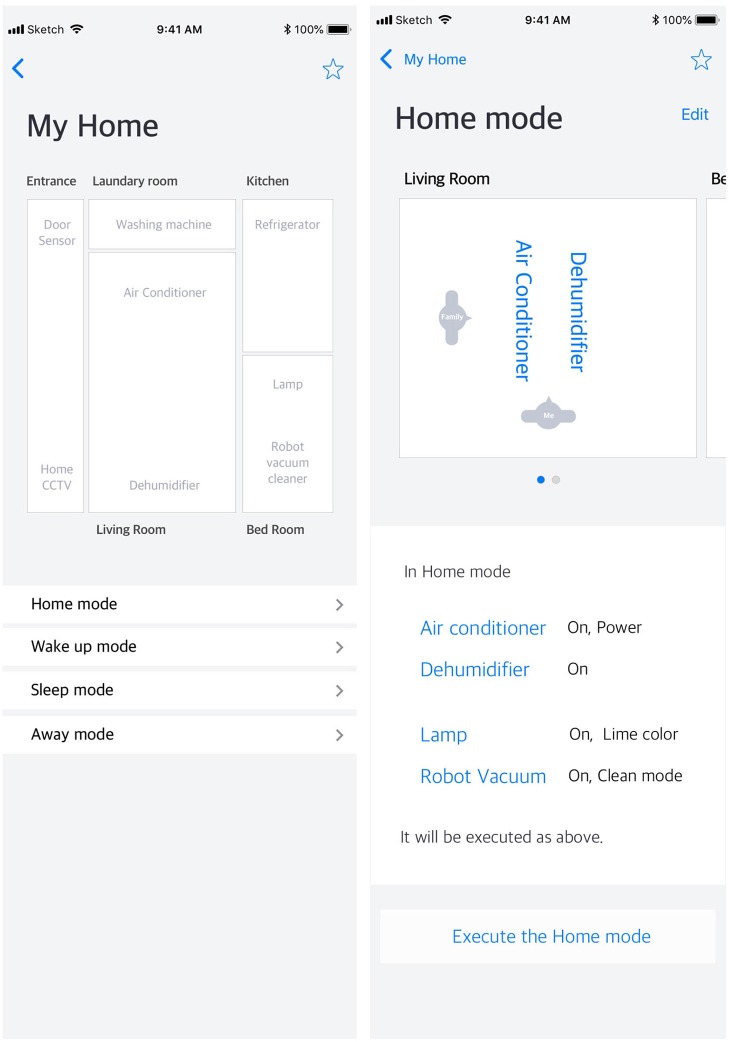
Screenshots of a mock smart home mobile application interface in Experiment 2. There were four different situation lists n the main page **(Left)** and there was information about the location, status, and context of the smart home devices in the sub-list page **(Right)**.

All phases were the same as in Experiment 1, except for Phase 3, where participants answered the SAGAT (Endsley, 1995, 2017; Scholtz et al., 2005) after watching videos of gesture interactions such as swiping and tapping on the mock smart home interface. To this end, we designed a mock smart home interface (see Figure 3) based on current smart home applications such as Amazon Echo, Google Home, Samsung Smart Things and LG IoT at Home. Key features of current market products, including situational modes with Home mode, Away mode, Awake mode, and Sleep mode, were included in the mock smart home interface. Thus, in the mock design interface, there were four different situation lists in the main page, and there was information about the location, status, and context of the smart home devices in the sub-list page.

Participants could watch, but not use, videos of someone using the mock-up mobile interface design of the smart home through the computer screen. This was intentional to avoid anticipated problems with participants using the mock smart home interface. It may have been difficult for participants to learn and become familiar with the smart home interface during the brief experiment. Considering the *gulf of execution*, referring to the difference between a user's expectation and the performance of the system (Norman, 1988, 1991), if the participant is a novice to that interface, the *gulf of execution* may be broadened.

Thus, we thought that watching the interaction video would be the ideal way to prevent participants from being negatively affected by the low usability of the smart home interface. In the recorded videos, a floating dot (a cursor on a personal computer) showed someone using the smart home interface and guided participants' perception. Watching such simulation videos (see Supplementary Materials) is a legitimate type of experimental stimulus (e.g., real interface, static interface image, simulation video) for the SAGAT (Endsley, 1995). Furthermore, the purpose of the SAGAT is not to measure the performance of the system but to evaluate one's understanding of the system interface and surrounding environment (Endsley, 1995). We thus thought it would be desirable to measure the situation awareness of the smart home interface through recorded video. Therefore, we made 16 videos in consideration of a 4 (situational mode: home, away, awake, sleep) × 2 (congruency with others' perspective: congruent, incongruent) × 2 (primary user-specific device: self, other) design. A participant watched eight videos randomly assigned to two situational modes among the 16 videos. Those eight videos were counterbalanced for preventing the order effect.

All participants responded to the nine questions of the SAGAT after each video ended. In SA Level 1, as the stage of perception for the interface, there were four questions about the location or existence of the device (e.g., “What devices are in the living room?”). To answer this question accurately, participants had to remember the top part of the mock smart home interface where the affordance of the co-user and location information of the devices were displayed. SA Level 2 was the stage of understanding the interface. Here, there were also four questions about the status and specific functions of that device (e.g., “What is the status of the lamp?”). All the contents related to SA Level 2 were located on the bottom of the interface. Finally, Level 3 was the projection stage based on understanding the interface, there projection-style questions (e.g., “Will the lamp turn on in the morning?”). A participant thus answered 72 questions evaluating situation awareness. All the SAGAT questions were multiple-choice and methods were referred to previous experiment (Scholtz et al., 2005).

#### Procedure

The overall experiment lasted ~30 min. The procedures of Phase 1 and Phase 2 were the same as in Experiment 1. In Phase 3, participants responded to the nine questions about the SAGAT after watching a video of someone using the mock smart home interface. Prior to the beginning of phase 3, participants were asked to watch thoroughly the contents of the video. Participants answered 72 SAGAT questions in total.

### Results and Discussion

Six participants below 90% accuracy in the memory task in Phase 2 were excluded from the data analysis. The data of 58 participants were used in the analysis.

**Figure 4:**
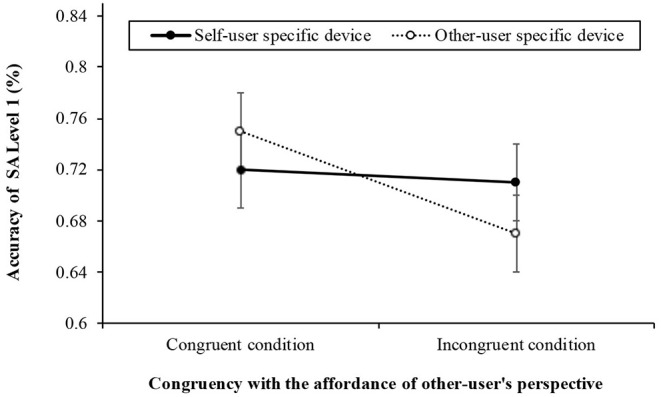
The results of accuracy (± *SE*) for answering the situation awareness (SA) Level 1 as the congruency with between the affordance of the other user's perspective and the word (2: congruent, incongruent) and the primary user-specific devices (2: self, other) in Experiment 2.

We conducted a 2 (congruency with others' perspective: congruent, incongruent) **×** 2 (primary user-specific device: self, other) repeated-measures ANOVA for the accuracy of SA Level 1, 2, and 3 in Phase 3 (see Table 4). The results of Level 1 (see Figure 4) revealed a significant difference [*F*_(1, 57)_ = 4.871, *p* = 0.031, η^2^p = 0.079] between the congruent (*M* = 0.73, *SD* = 0.18) and incongruent conditions (*M* = 0.69, *SD* = 0.18) in the affordance of the other user's perspective and the direction of the device. However, there was neither a significant difference for primary user-specific device [*F*_(1, 57)_ = 0.225, *p* = 0.637, η^2^p = 0.004], nor was there a significant interaction [*F*_(1, 57)_ = 2.694, *p* = 0.106, η^2^p = 0.045] between congruency and primary user-specific device.

**Table 4:** A 2 (congruency with others' perspective: congruent, incongruent) × 2 (primary user-specific device: self-user, other-user) repeated-measures ANOVA in Experiment 2.

**Source**	**df**	***F***	**η^2^**	***p***
Congruency	1	4.871	0.79	0.031
Primary user-specific device	1	0.225	0.004	0.637
Error	57	(0.022)		
Congruency **×** primary user-specific device	1	2.694	0.045	0.106
Error	57	(0.026)		

*Values enclosed in parentheses represent mean square errors*.

**Figure 5:**
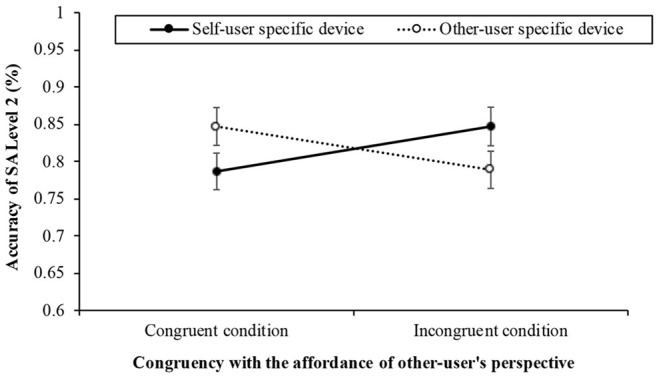
The results of accuracy (± *SE*) for answering the situation awareness (SA) Level 2 as the congruency with between the affordance of the other user's perspective and the word (2: congruent, incongruent) and the primary user-specific devices (2: self, other) in Experiment 2.

The Level 2 results (see Figure 5) revealed neither a significant difference in congruency [*F*_(1, 57)_ = 4.871, *p* = 0.949, η^2^p = 0.000] nor primary user-specific device [*F*_(1, 57)_ = 0.002, *p* = 0.962, η^2^p = 0.000] but there was a significant interaction effect [*F*_(1, 57)_ = 4.767, *p* = 0.033, η^2^p = 0.077] between congruency and primary user-specific device. However, in self-user specific device condition, the Bonferroni multiple pairwise comparison showed that there was a marginally significant difference between congruent condition (*M* = 0.79, *SD* = 0.19) and incongruent condition (*M* = 0.85, *SD* = 0.20, *p* = 0.097) in self-specific devices condition. On the other hand, the Bonferroni multiple pairwise comparison showed not a significantly difference between two conditions that the other-user specific device condition and congruent condition (*M* = 0.85, *SD* = 0.19) and incongruent condition (*M* = 0.79, *SD* = 0.19, *p* = 0.120).

The Level 3 results showed no significant difference based on congruency [*F*_(1, 57)_ = 1.425, *p* = 0.237, η^2^p = 0.024], primary user-specific device [*F*_(1, 57)_ = 0.054, *p* = 0.817, η^2^p = 0.001], or the interaction effect [*F*_(1, 57)_ = 2.254, *p* = 0.139, η^2^p = 0.038]. We thus found that when we predict how the smart home device works, adopting the affordance of other users in the smart home interface may not be helpful.

Counter to the results of Experiment 1, we found the congruency effect wherein participants in the congruent condition adopted the affordance of the other user's perspective when perceiving (i.e., SA Level 1) the smart home interface than did those in the incongruent condition. In Level 1, participants should retrieve the affordance information co-users and the device name displayed on the top part of the smart home interface. The information on the top part of the smart home interface was the same as the stimulus image in Experiment 1 (see Figures 1, 3 right panel). Even though the same stimulus was used, we confirmed there was no congruency effect in Experiment 1, but there was a congruency effect in SA Level 1 of Experiment 2. This is because the experimental task differed between the two experiments. For example, participants should answer who the primary user of that smart home device is (i.e., semantic categorization task) in Experiment 1, while participants were requested to answer where the device is located in Experiment 2. In Experiment 2, the SAGAT might have inhibited the category effect that emerged in Experiment 1. In addition, the complexity of the stimulus in Experiment 2 was relatively increased as compared to Experiment 1. Thus, it may have contributed to recognizing affordances that are usually processed implicitly (Baber, 2018) in Experiment 2.

We found the interaction effect between congruency and primary user specific device in SA Level 2. However, it was a just marginally significant difference between congruent and incongruent conditions only for the self-user specific device. The result showed an inverted congruency effect that the incongruent condition was more accurate than the congruent condition. Even though it is marginally effective, this result may suggest that the inclusion of human figures affordance on the interface may impair situation awareness at least self-specific device and congruent condition.

In addition, given that SA Level 2 measures how well participants understand information about the specific configuration or status of the devices written on the interface, they may tend to try to take their viewpoints when participants read information about their devices (i.e., self-user specific device). If so, the self-user affordance currently in sight may remind them of the specific information of the smart home interaction associated with that affordance. Thus, we should consider as an important factor whether the device is frequently used by the user or not in the understanding stage of SA (i.e., SA Level 2).

In Level 3, there was no significant difference in any conditions at the projection stage of SA. Given that higher SA phases are associated with greater mental effort (Endsley, 1995), these results imply that VSP taking may occur only in lower SA such as in the perception phase, but not in higher SA phases such as projection. In conclusion, the results of Experiment 2 revealed that VSP taking helped for SA Levels 1 in the smart home interface. In addition, we confirmed that a natural phenomenon in social interaction—adopting another person's perspective—can occur not only in a virtual environment but may also help to enhance situation awareness about the smart home interface.

## General Discussion

The current study examined whether spontaneous VSP taking can occur in a virtual environment similar to an actual smart home interface. Moreover, we assessed whether spontaneous VSP taking enhances situational awareness about the smart home interface. We revealed that if the affordance of another person is displayed on the smart home interface, participants have the propensity to adopt the other's perspective.

The novelty of the current study is that we confirmed that even in the smart home interface, a virtual environment, we still have the propensity to adopt the perspective of others. Considerable research has shown that we adopt others' perspectives as a result of the mere presence of others in a shared physical realm (Tversky and Hard, 2009; Kockler et al., 2010; Freundlieb et al., 2016; Furlanetto et al., 2016; Cavallo et al., 2017; Quesque et al., 2018). This propensity may be extended to the realm of mental activity, such as thinking and reading (Freundlieb et al., 2018), and even agentic features of inanimate objects such as an arrow (Zwickel, 2009; Heyes, 2014; Santiesteban et al., 2014). This phenomenon may thus be essential to successful social interaction or communication by adopting others' perspectives (Quesque et al., 2018). It may then be no surprise that we showed VSP taking, which usually occurs in real social interaction, in the smart home interface.

In addition, we found that VSP taking can affect mental activity such as situation awareness, which is necessary to understand the interface of the system. This means that VSP taking can also occur in other mental activities, such as situation awareness, in addition to reading, which is a recent finding in VSP taking research (Freundlieb et al., 2018). To this end, we constructed a mock-up design of a smart home interface referring to that previous experiment (Freundlieb et al., 2018). We replicated the previous result that participants adopt others' perspectives in mental activity in SA Levels 1 in Experiment 2. We not only replicated the previous result but found that VSP taking also occurs in another mental activity context: situation awareness. VSP taking also enhances situation awareness about the smart home interface.

The current findings have useful implications for designing smart home interfaces. First, the affordances of co-users, which induce the VSP taking phenomenon, can be effective in designing IoT interactions. The current study attempted to use a previous experiment to improve the validity of the current experiment. The boundary conditions of the experiment were the affordance of the self, the affordance of the other, and the device words located in the middle of the stimulus, which was the same stimulus image as the previous experiment. Given that VSP taking did not occur in a spatial memory-based task (Kelly et al., 2018), it is important that the affordance of co-users is always displayed in a smart home controller for effective interface design. In addition, the human figure affordance (Tarampi et al., 2016) is suitable for mental simulation to deal with information such as SA Level 1.

We added location information on where the smart home device is in the current experiment. For example, we labeled the top of that stimulus image as living room, kitchen, bedroom, and so on. This suggests that a room-based interface design can be effective in the smart home interface. This is important not only because rooms may be one of the most intuitive large-scale categories for classifying many devices, but also it might contribute to enhancing situation awareness by indicating the shared physical context. Of course, further development in the future is necessary because the room layout used in the current mock smart home interface was relatively artificial and superficial.

There were a few limitations, as this study was the first attempt to apply both VSP taking and situation awareness to the smart home interface. We restricted the number of other users to only one person, and this was our intentional choice to examine whether VSP taking would occur. It was not reflected that there might be multiple users that share the device rather than just two users in an actual smart home context. Future studies thus need to examine not only whether VSP taking also occurs in the smart home interface where more than two people co-occur but also that the phenomenon still helps to enhance situation awareness. Given that affordances still require cognitive resources (Grgic et al., 2016), increasing the number of the affordances may lead to an overly heavy load for users to perceive and understand complex information about the smart home interface.

In the results of SA Level 2 of Experiment 2, we found that the possibility that participant's situation awareness is impaired if human figure affordance is displayed for the device mainly using by self-user. This is because there was a marginally significant inverted congruency effect for self-user specific device. If future research reveals that SA Level 2 results are robust, we should consider why such an inverted congruency effect appears only for the self-user specific device. We think that one of the experiment designs to find the reason is to exclude self-user affordance on the interface. From the participant's point of view, there is one human (i.e., confederate) in the experiment of VSP taking in the physical space. However, there are two human figures (i.e., self-user affordance and other-user affordance) in the experiment of VSP taking on the smart home interface. Therefore, if the self-user affordance is removed on the interface, the inverted congruency effect for self-user specific device in SA Level 2 may disappear. Also, we think that it may be necessary to add a control condition without a human agent affordance to compare conventional devices and to eliminate a possibility that the presence of a human figure has a negative effect on overall situational awareness.

Furthermore, future research is needed to generalize the results of this research. Most of the participants in this study are college students in psychology and live in South Korea. The diversity of the participants needs to be pursued to see if the results of this study are repeated without being limited to these age groups and regions. In addition, it may be necessary to increase the degree of difficulty of device information and to develop a non-memory based experimental task (i.e., Phase 1 and Phase 2) that reflects the real situation. The result of the current study implies that the affordance of other users helps the retrieval of device information. Even if errors occur unexpectedly, if affordances can help the user retrieve device information or solve the problem, we believe that the affordance of the user might be one of the most effective design strategies in IoT interaction.

## Data Availability Statement

The datasets generated for this study are available on request to the corresponding author.

## Ethics Statement

The studies involving human participants were reviewed and approved by Institutional Review Board of Yonsei University. The patients/participants provided their written informed consent to participate in this study.

## Author Contributions

SY developed the study concept, design, and performed data collection and analysis under the supervision of KH.

### Conflict of Interest

The authors declare that the research was conducted in the absence of any commercial or financial relationships that could be construed as a potential conflict of interest.

## Supplementary Material

Supplementary Video 1 This is a video that was presented as a stimulus to the participants in Experiment 2.
